# Supplementary material for: Structural basis of soluble membrane attack complex packaging for clearance
Source: Nat Commun. 2021 Oct 19;12:6086. doi: 10.1038/s41467-021-26366-w (PMC8526713; doi:10.1038/s41467-021-26366-w)
Supplement: Supplementary file 1 — Supplementary Information [file 41467_2021_26366_MOESM1_ESM.pdf]

Structural basis of soluble membrane attack complex packaging for clearance

Anaïs Menny[1#], Marie V. Lukassen[2,3#], Emma C. Couves[1], Vojtech Franc[2,3], Albert J.R. Heck[2,3], Doryen Bubeck[1*]

[1] Department of Life Sciences, Sir Ernst Chain Building, Imperial College London, London SW7 2AZ, United Kingdom

[2] Biomolecular Mass Spectrometry and Proteomics, Bijvoet Center for Biomolecular Research and Utrecht Institute for Pharmaceutical Sciences, Utrecht University, Padulaan 8, 3584 CH Utrecht, The Netherlands

[3] Netherlands Proteomics Center, Padulaan 8, 3584 CH Utrecht, The Netherlands

[#] Equal contribution

*Correspondence to: d.bubeck@imperial.ac.uk

ONLINE SUPPLEMENTARY INFORMATION

SUPPLEMENTARY TABLES

Supplementary Table 1.

Cryo-EM data collection, refinement, and validation statistics

	#1 1C9-sMAC (EMD-12649)	#2 2C9-sMAC (EMD-12651) (PDB 7NYD)	#3 3C9-sMAC (EMD-12650) (PDB 7NYC)	#4 2C9-sMAC^{stalk} (EMD-12648) (PDB 7NYD)	#5 2C9-sMAC^{arc} (EMD-12647) (PDB 7NYD)	#6 3C9-sMAC^{arc} (EMD-12646) (PDB 7NYC)
**Data collection and processing**						
Magnification	130k	130k	130k	130k	130k	130k
Voltage (kV)	300	300	300	300	300	300
Electron exposure (e–/Å^2)	40 D_1 42 D_2	40 D_1 42 D_2	40 D_1 42 D_2	40 D_1 42 D_2	40 D_1 42 D_2	40 D_1 42 D_2
Defocus range (μm)	-1.1 to -2.3 D_1 , -1.1 to -2.1 D_2	-1.1 to -2.3 D_1 , -1.1 to -2.1 D_2	-1.1 to -2.3 D_1 , -1.1 to -2.1 D_2	-1.1 to -2.3 D_1 , -1.1 to -2.1 D_2	-1.1 to -2.3 D_1 , -1.1 to -2.1 D_2	-1.1 to -2.3 D_1 , -1.1 to -2.1 D_2
Pixel size (Å)	1.047 D_1 1.048 D_2	1.047 D_1 1.048 D_2	1.047 D_1 1.048 D_2	1.047 D_1 1.048 D_2	1.047 D_1 1.048 D_2	1.047 D_1 1.048 D_2
Symmetry imposed	None	None	None	None	None	None
Initial particle images (no.)	1,084,293 D_1 195,552 D_2	1,084,293 D_1 195,552 D_2	1,084,293 D_1 195,552 D_2	1,084,293 D_1 195,552 D_2	1,084,293 D_1 195,552 D_2	1,084,293 D_1 195,552 D_2
Final particle images (no.)	99,277	142,499	85,151	142,499	96,118	85,151
Map resolution (Å) FSC threshold 0.143	3.8	3.3	3.5	3.6	3.3	3.8
Map resolution range (Å)	3.6-9.1	3.0-10.9	3.2-11.1	3.4-7.7	3.2-6.8	3.6-9.1
**Refinement**						
Initial model used (PDB code)		6H04, 4A5W, 2WCY, 6H03, 6CXO	6H04, 4A5W, 2WCY, 6H03, 6CXO	6H04, 4A5W, 2WCY, 6H03, 6CXO	6H04, 4A5W, 2WCY, 6H03, 6CXO	6H04, 4A5W, 2WCY, 6H03, 6CXO
Model resolution (Å) FSC threshold 0.5		3.2	3.5	3.2	3.2	3.5
Model resolution range (Å)						
Map sharpening *B* factor (Å^2)	Locally sharpened	Locally sharpened	Locally sharpened	Locally sharpened	Locally sharpened	Locally sharpened
Model composition						
Non-hydrogen atoms		38180	41,094	38,180	38,180	41,094
Protein residues		4,940	5,312	4,940	4,940	5,312
Ligands						
B factors (Å^2)						
Protein		88	98	88	88	98
Ligand						
R.m.s. deviations						
Bond lengths (Å)		0.012	0.013	0.012	0.012	0.013
Bond angles (°)		1.917	1.984	1.917	1.917	1.984
Validation						
		1.28	1.34	1.28	1.28	1.34

MolProbity score	1.84	2.31	1.84	1.84	2.31
Clashscore	0.49	0.68	0.49	0.49	0.68
Poor rotamers (%)					
Ramachandran plot					
Favored (%)	95.17	95.19	95.17	95.17	95.19
Allowed (%)	4.81	4.81	4.81	4.81	4.81
Disallowed (%)	0.02	0.00	0.02	0.02	0.00

D_1 and D_2 refer to the two datasets which were combined for image processing. D_1 was collect at 0 degrees and D_2 was taken at a tilt angle of 37 degrees.

1C9-sMAC, 2C9-sMAC, and 3C9-sMAC maps refer to the sMAC reconstructions after 3D classification, in which different stoichiometries of C9 in complex were separated. 2C9-sMAC^{stalk} is the 2C9-sMAC map after density subtraction and focused refinement on C5b and the C-terminal domains of C7. 2C9-sMAC^{arc} refers to the 2C9-sMAC map after density subtraction and focused refinement on the C9 oligomer. 3C9-sMAC^{arc} refers to the 3C9-sMAC map after density subtraction and focused refinement on the C9 oligomer.

34 SUPPLEMENTARY FIGURES

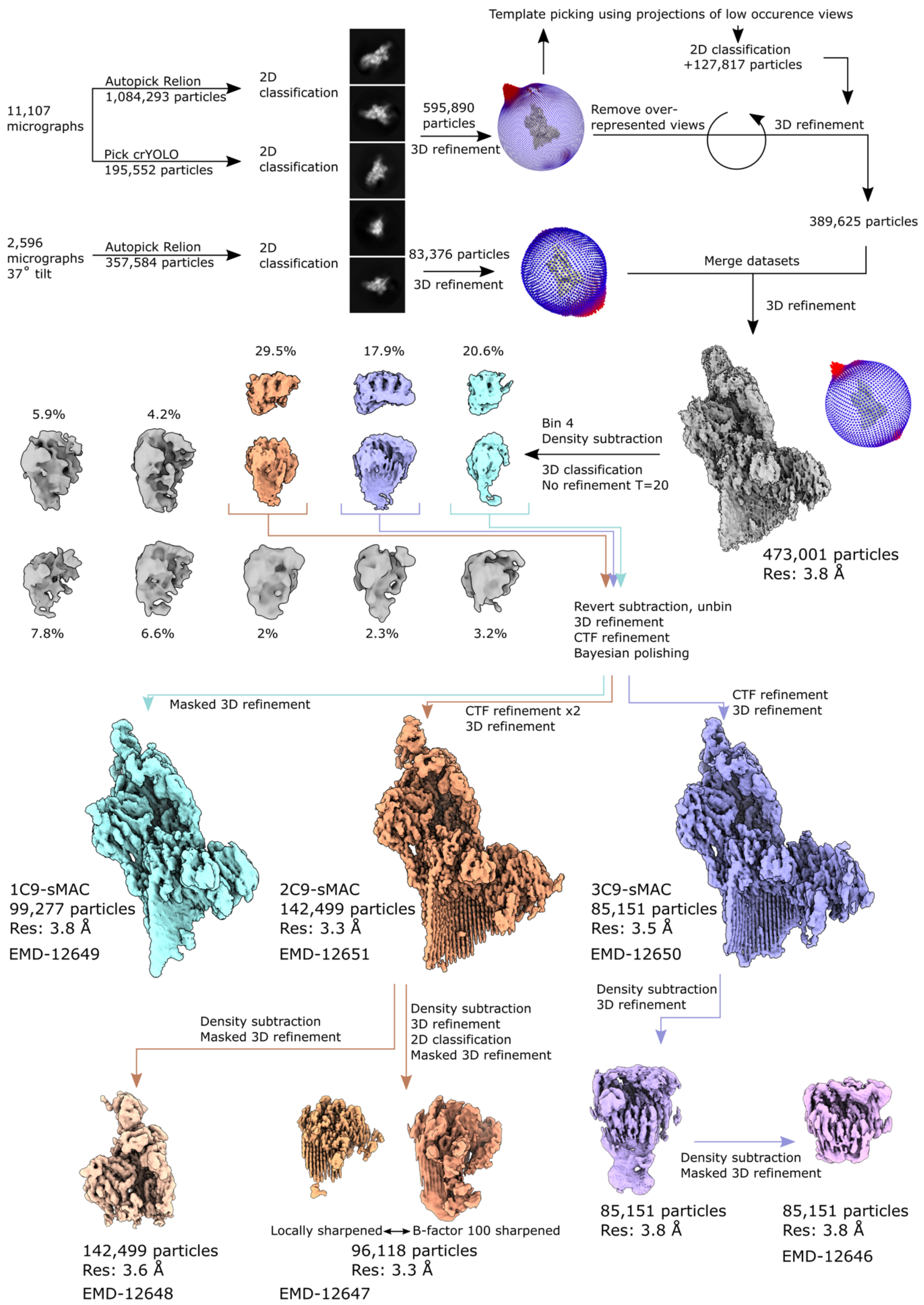

35

Supplementary Figure 1: CryoEM image processing workflow. Particles were picked from micrographs using autopicking programs in Relion and cryYOLO to account for differences in ice-thickness across the micrographs. Images were subject to 2D classification and an initial auto-refinement, resulting in a reconstruction with strong preferred orientations. To improve the angular distribution of the dataset, we combined three strategies: 1) We implemented template-based picking procedures using projections of low occurrence views. 2) We pruned particles from over-represented views. 3) We incorporated an additional dataset collected at a tilt angle of 37°. Duplicate particles were removed and datasets were merged. Data was subject to an additional 3D auto-refinement to generate a consensus sMAC reconstruction with an improved angular distribution. To separate sMAC maps that contained different numbers of C9 molecules we subtracted density corresponding to the core complement complex (C5b6, C7, C8) and used 3D classification with no refinement. Maps with clear density for either 1 (cyan), 2 (orange) or 3 (purple) copies of C9 were taken forward. Reconstructions for each of these three classes were then calculated based on the corresponding particles before density subtraction. Data was subjected to Bayesian polishing and multiple rounds of per-particle CTF refinement before a final 3D auto-refinement to generate the sMAC maps: 1C9-sMAC (3.8 Å) (EMD-12649), 2C9-sMAC (3.3 Å) (EMD-12651), and 3C9-sMAC (3.5 Å) (EMD-12650). To better resolve density above the C9 LDL domains, we used particles corresponding to the 3C9-sMAC map (purple) and subtracted density for the core complement complex. The resulting reconstruction (light purple) showed improved density in this region. We next performed an additional density subtraction and focused our 3D refinement on the core of C9 to generate the C9-clusterin focus-refined 3C9-sMAC map (pink, 3.8 Å) (EMD-12646). To better resolve density corresponding to C5b, we used particles

corresponding to the 2C9-sMAC map (orange) and subtracted density for the MACPF arc. Using a masked 3D auto-refinement we calculated a map corresponding to C5b and C6/C7 C-terminal domains (tan, 3.6 Å) (EMD-12648). To better resolve the alternative conformation of C9 in sMAC, we used particles corresponding to the 2C9-sMAC map (orange) and subtracted density for the core complement complex. Particles were subjected to a further 2D classification to improve homogeneity of the population and a final reconstruction of the C9 focus-refined map was calculated (light orange, 3.3 Å) (EMD-12647). The locally sharpened map was used to build the intermediate C9 conformation in sMAC. Blurring the map using an ad hoc B-factor (100), we observe density in the vicinity of the hydrophobic β-hairpins of the C9 MACPF, which may correspond to vitronectin.

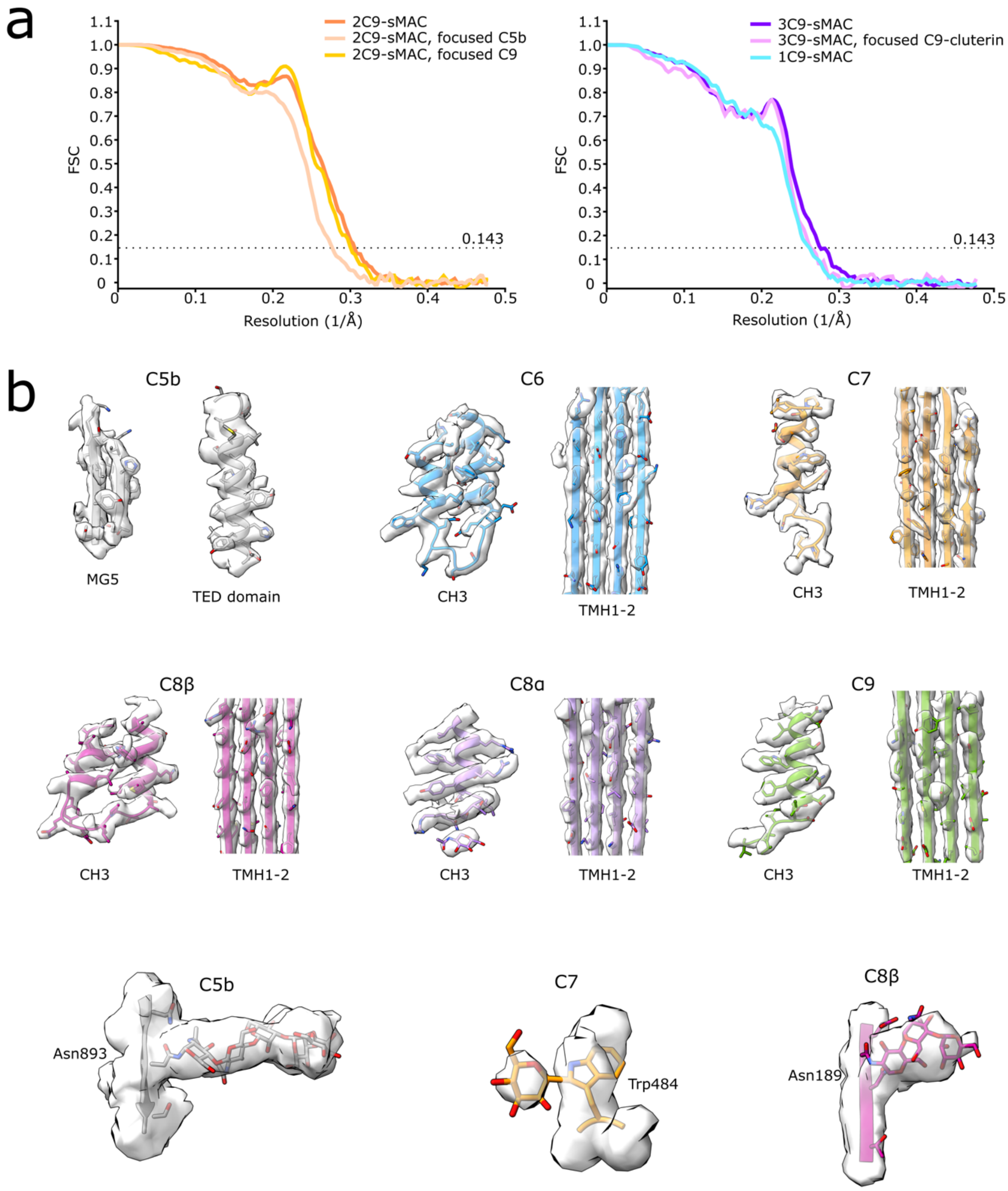

Supplementary Figure 2: CryoEM map validation. (a) Fourier Shell Correlation (FSC) curves for all deposited maps: 1C9-sMAC (EMD-12649), 2C9-sMAC (EMD-12651), 3C9-sMAC (EMD-12650), C5b-focused 2C9-sMAC (EMD-12648), C9-focused 2C9-sMAC (EMD-12647), and C9-clusterin focused 3C9-sMAC (EMD-12646). (b) cryoEM density maps (transparent surface) overlaid with atomic models for complement

proteins (colored according to protein composition). Representative densities for modeled glycans are shown in the bottom panel.

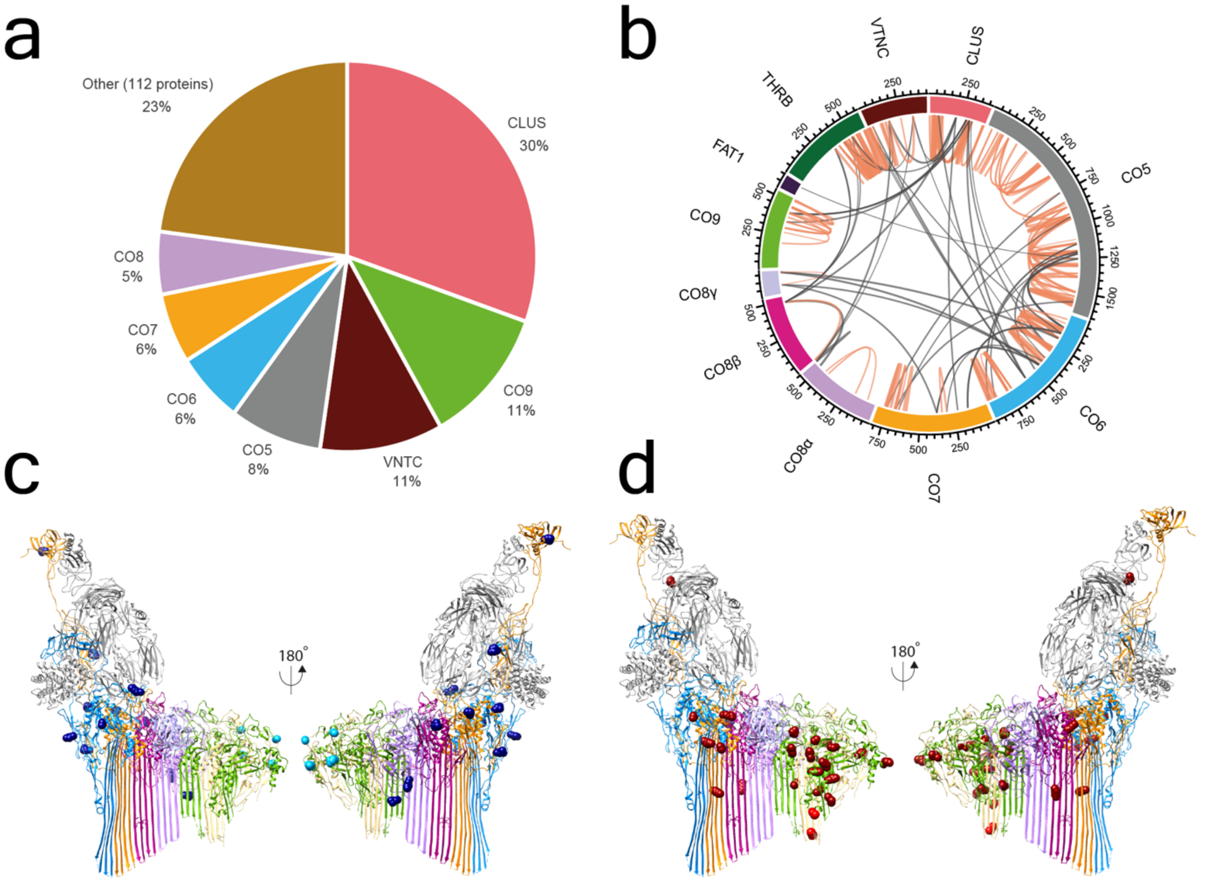

Supplementary Figure 3: Chaperone interactions of sMAC. (a) Label-free quantification (iBAQ) of proteins in the sMAC sample by LC-MS/MS. (b) sMAC circos plot of identified disuccinimidyl sulfoxide (DSS) cross-links. The complement components are cross-linked to thrombin (THRB), cadherin 8 domain of protocadherin FAT1 (FAT1) and the chaperones vitronectin (VTNC) and clusterin (CLUS). Intra-links are shown as orange lines and inter-links are shown as black lines. (c) Residues of sMAC uniquely cross-linked to vitronectin are shown as dark blue spheres plotted on the 3C9-sMAC model. Light blue spheres are not resolved in all three C9 molecules. (d) Residues of sMAC uniquely cross-linked to clusterin are shown as dark red

spheres plotted on the 3C9-sMAC model. Residues that are not resolved in all three C9 molecules are colored light red. For panels c and d complement proteins are colored as in Fig. 1: C5b (grey); C6 (blue); C7 (orange); C8α (light purple); C8β (dark purple); C8γ (lilac); C9 (alternating green and tan). For panels b-d source data are provided as a Source Data file.

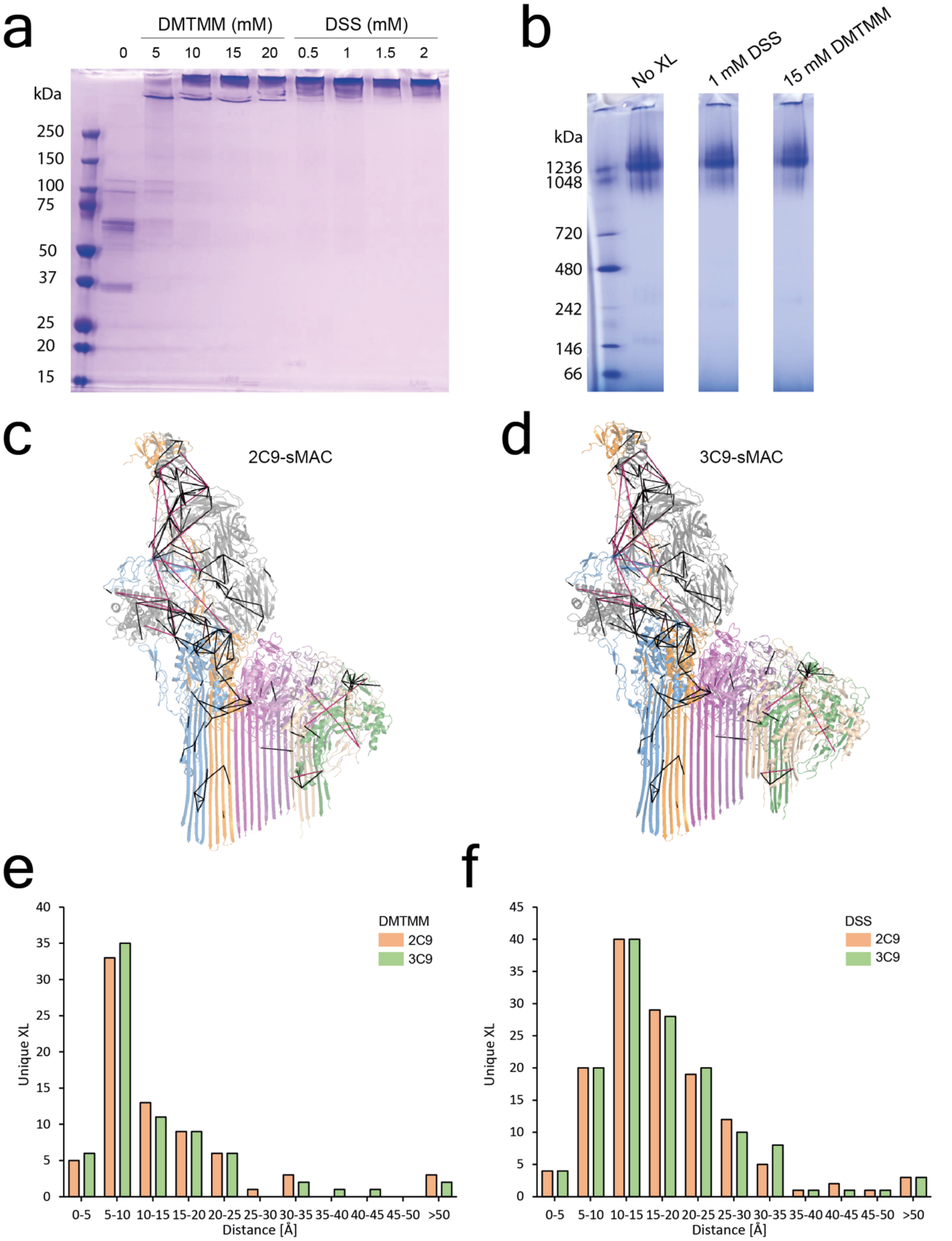

Supplementary Figure 4: Cross-linker optimization and mapping of identified cross-links on structural models of sMAC. (a) Sodium dodecyl sulfate polyacrylamide gel

electrophoresis (SDS-PAGE) was used to determine optimal cross-linker concentration. sMAC (10 μg) was cross-linked with 4-(4,6-Dimethoxy-1,3,5-triazin-2-yl)-4-methylmorpholinium chloride (DMTMM) (5-20 mM) or disuccinimidyl sulfoxide (DSS) (0.5-2 mM) and quenched before running on the SDS-PAGE (b) Blue native-PAGE (BN-PAGE) of cross-linked sMAC (10 μg) using optimal DSS (1 mM) or DMTMM (15 mM) concentrations. sMAC sample with no crosslinker present (no XL) is also indicated for reference. The gels in panels a-b were repeated twice with similar results. (c-d) Identified cross-links plotted on the sMAC structural models containing (c) two C9 or (d) three C9 molecules. Cross-links below the distance restraints (DSS <30Å and DMTMM < 20Å) are shown as black lines and distances above the restraints are shown as pink lines. Complement proteins are colored as in Fig. 1: C5b (grey); C6 (blue); C7 (orange); C8α (light purple); C8β (dark purple); C8γ (lilac); C9 (alternating green and tan). (e-f) Histogram of Cα-Cα distances of (e) DMTMM and (f) DSS cross-links mapped on the 2C9- and 3C9-sMAC atomic models. Displayed cross-links were identified in all three replicates. For panels c-f source data are provided as a Source Data file.

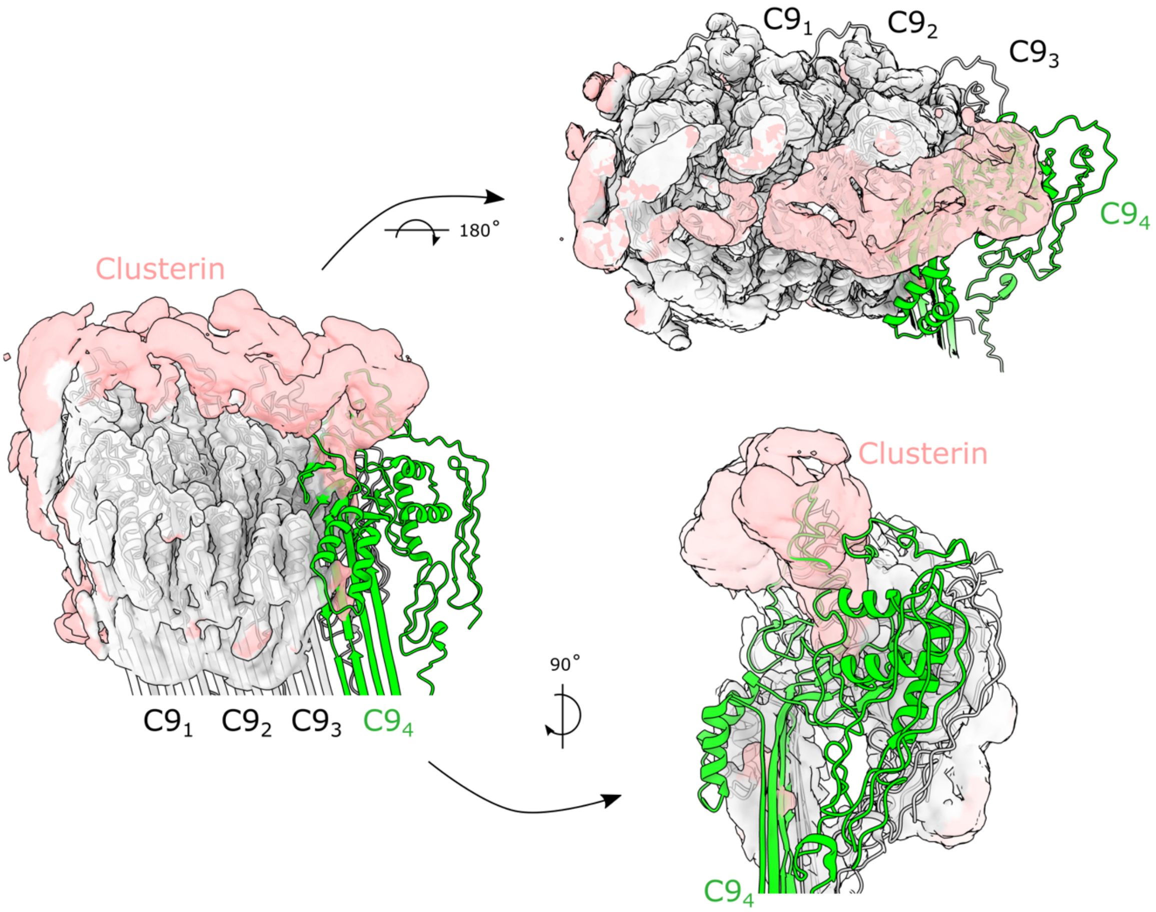

Supplementary Figure 5: Clusterin sterically blocks C9 oligomerization. An oligomer comprised of four C9 molecules was extracted from the MAC structure (PDB ID: 6H04) and fit into the EM density of the 3C9 focus refined map (transparent surface). While the first three C9 molecules ($C9_1$, $C9_2$, $C9_3$: grey ribbons) fit the density occupied by C9 in sMAC (grey transparent surface), the fourth C9 molecule ($C9_4$: green ribbons) clashes with density occupied by clusterin in sMAC (pink transparent surface).

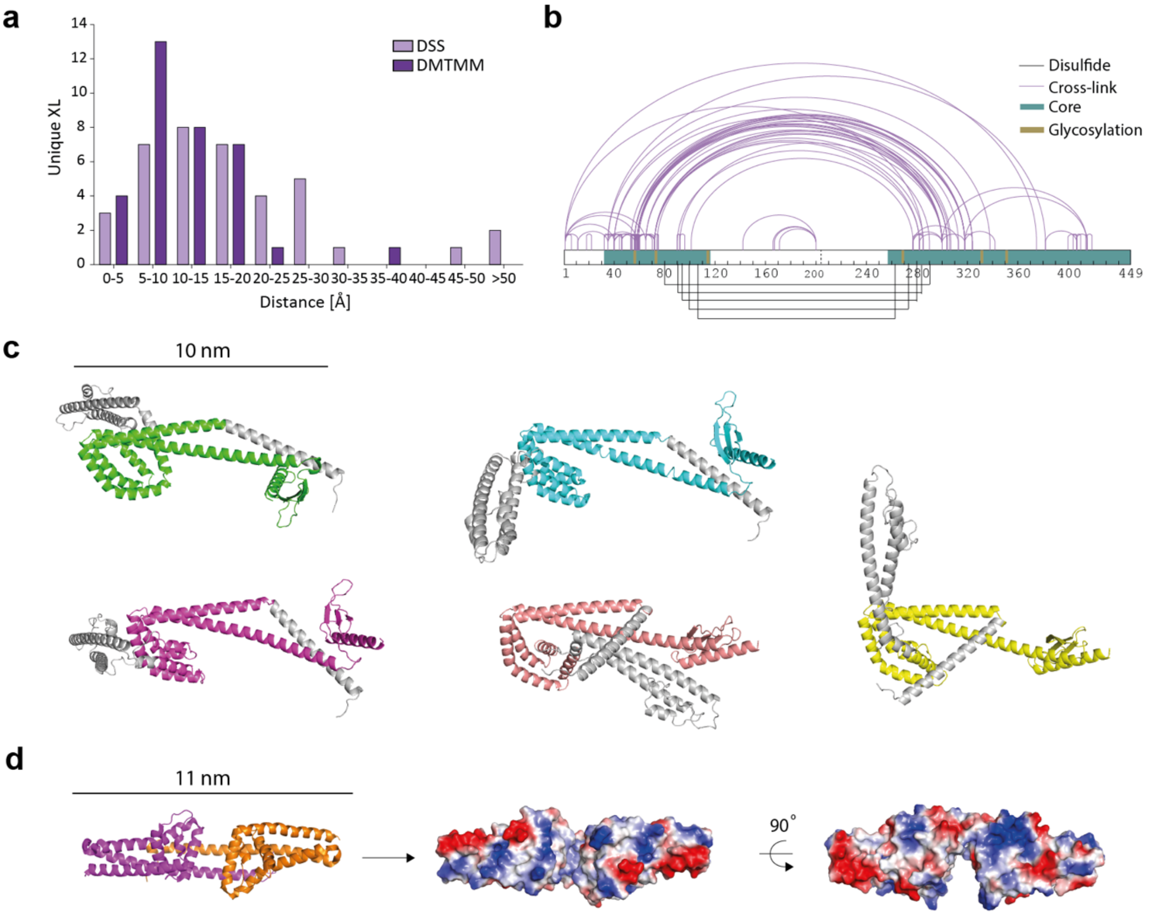

Supplementary Figure 6: Structural model for clusterin. (a) Histogram of Cα-Cα distances of disuccinimidyl sulfoxide (DSS, light purple) and 4-(4,6-Dimethoxy-1,3,5-triazin-2-yl)-4-methylmorpholinium chloride (DMTMM, dark purple) cross-links plotted on the clusterin core model. (b) Identified clusterin inter-links are mapped onto the clusterin sequence shown with purple lines. The five disulfide bridges are shown as black lines. The common core is colored teal and reported glycosylation sites are marked in sand. For panels a-b source data are provided as a Source Data file. (c) Panel of structural models generated by trRosetta. The consensus core region is uniquely colored by model, while long helical extensions that flexibly hinge from the core are grey. (d) Structure of dimeric CspA from *Borrelia burgdoferi* (PDB ID: 1W33) shown as ribbons where each monomer is individually colored (purple and orange,

respectively) (left panel). Middle and right panels show the Coulombic electrostatic potential of the CspA dimer ranging from -64 (red) to 64 (blue) kcal/(mol·e).

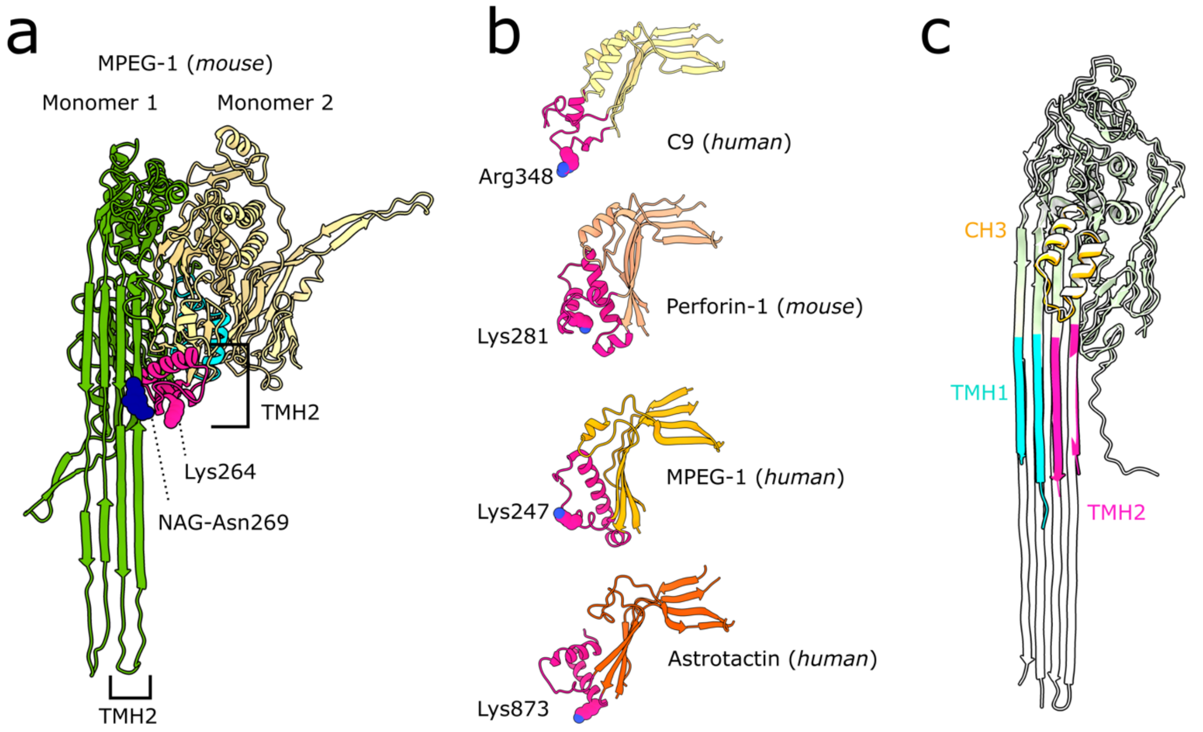

Supplementary Figure 7: MACPF structural transitions. (a) Model of a potential intermediate confirmation of macrophage expressed gene 1 (MPEG-1) generated by superposing a single monomer from a soluble conformation (PDB ID: 6SB3) onto the MPEG-1 pore structure (PDB ID: 6SB5). Two adjacent MPEG-1 monomers are shown. Monomer 1 (green) is from the transmembrane conformation. Monomer 2 (yellow) is the superposed soluble conformation. This superposition places a positively charged residue (Lys264, pink surface) within TMH2 of monomer 2 (pink) near a glycan (blue surface) on the extended β-hairpins of the preceding monomer (monomer 1, green). TMH1 of the soluble monomer (cyan) is shown for reference. (b) Central kinked β-sheet and TMH2 helical bundle of the MACPF domains of C9 (intermediate conformation in sMAC), perforin-1 (PDB ID: 3NSJ), MPEG-1 (PDB ID: 4OEJ) and astrotactin-2 (PDB ID: 5J68). In each case, a similarly oriented positively charged

residue with TMH2 is highlighted (spheres). TMH2s are indicated in pink; TMH1s are removed for clarity. (c) Superposition of the transmembrane conformation of C9 as seen in MAC (white, PDB ID: 6H03) with the penultimate C9 in 2C9-sMAC (colored ribbons: CH3 is yellow; TMH1 is cyan; TMH2 is pink; the remainder of C9 is green).

SOURCE DATA

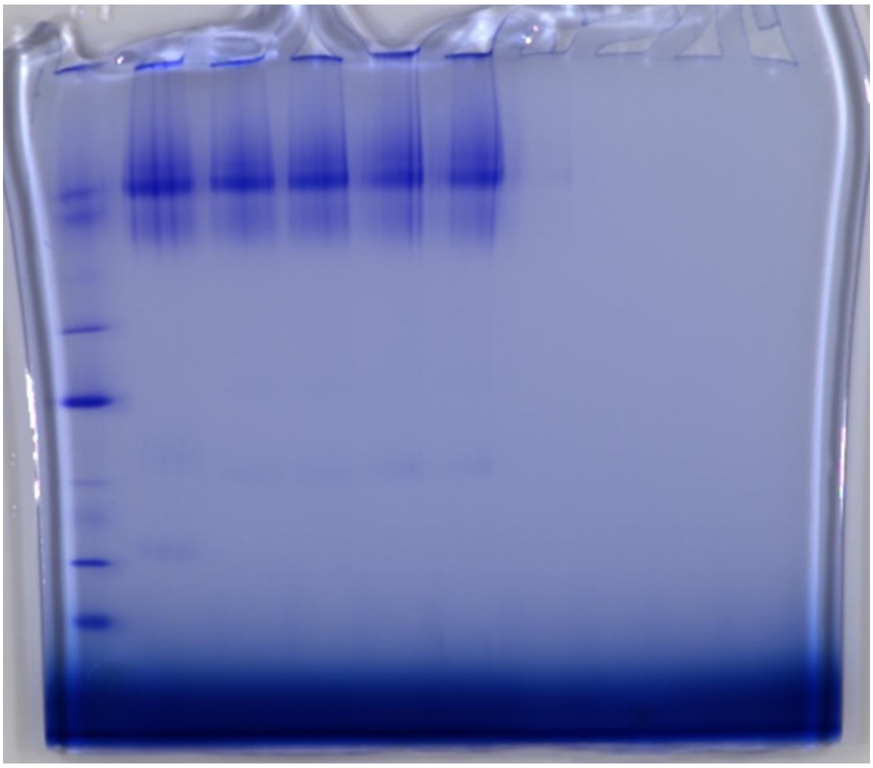

Supplementary Figure 4b: Lane 1 is the marker, lane 2 is the sMAC sample with no crosslinker added, lane 3 is the sMAC sample with the addition of 0.5 mM DSS, lane 4 is the sMAC sample with the addition of 1 mM DSS, lane 5 is the sMAC sample with the addition of 10 mM DMTMM, lane 6 is the sMAC sample with the addition of 15 mM DMTMM.

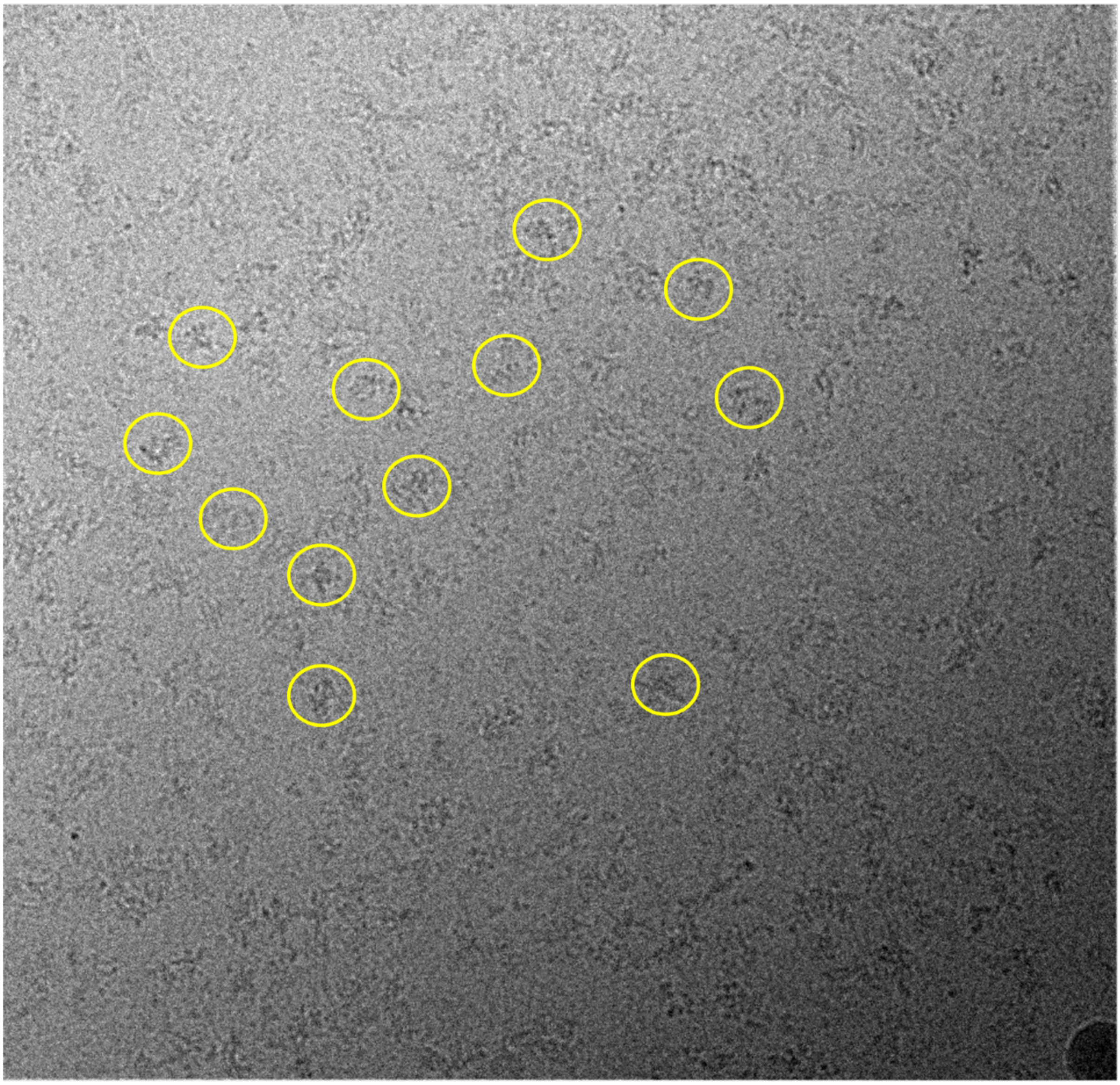

Supplementary Figure 2: Representative raw electron micrograph from the data set underlying the cryo EM maps in Supplementary Figure 2. Representative individual particles are circled.
