# Supplementary material for: Structural basis of soluble membrane attack complex packaging for clearance
Source: Nat Commun. 2021 Oct 19;12:6086. doi: 10.1038/s41467-021-26366-w (PMC8526713; doi:10.1038/s41467-021-26366-w)
Supplement: Supplementary file 3 — Description of Additional Supplementary Files [file 41467_2021_26366_MOESM3_ESM.pdf]

Description of Additional Supplementary Files

File name: Supplementary Data 1

Description: File that contains the output of the mass spectrometry identification software.
