# Supplementary material for: Structural basis of soluble membrane attack complex packaging for clearance
Source: Nat Commun. 2021 Oct 19;12:6086. doi: 10.1038/s41467-021-26366-w (PMC8526713; doi:10.1038/s41467-021-26366-w)
Supplement: Supplementary file 5 — Reporting Summary [file 41467_2021_26366_MOESM5_ESM.pdf]

nature research

Corresponding author(s): Doryen Bubeck

Last updated by author(s): Sep 22, 2021

Reporting Summary

Nature Research wishes to improve the reproducibility of the work that we publish. This form provides structure for consistency and transparency in reporting. For further information on Nature Research policies, see our Editorial Policies and the Editorial Policy Checklist.

Statistics

For all statistical analyses, confirm that the following items are present in the figure legend, table legend, main text, or Methods section.

n/a	Confirmed	
☐	☒	The exact sample size (*n*) for each experimental group/condition, given as a discrete number and unit of measurement
☐	☒	A statement on whether measurements were taken from distinct samples or whether the same sample was measured repeatedly
☒	☐	The statistical test(s) used AND whether they are one- or two-sided<br>*Only common tests should be described solely by name; describe more complex techniques in the Methods section.*
☒	☐	A description of all covariates tested
☒	☐	A description of any assumptions or corrections, such as tests of normality and adjustment for multiple comparisons
☒	☐	A full description of the statistical parameters including central tendency (e.g. means) or other basic estimates (e.g. regression coefficient) AND variation (e.g. standard deviation) or associated estimates of uncertainty (e.g. confidence intervals)
☒	☐	For null hypothesis testing, the test statistic (e.g. F, t, r) with confidence intervals, effect sizes, degrees of freedom and P value noted<br>*Give P values as exact values whenever suitable.*
☒	☐	For Bayesian analysis, information on the choice of priors and Markov chain Monte Carlo settings
☒	☐	For hierarchical and complex designs, identification of the appropriate level for tests and full reporting of outcomes
☒	☐	Estimates of effect sizes (e.g. Cohen's *d*, Pearson's *r*), indicating how they were calculated

Our web collection on statistics for biologists contains articles on many of the points above.

Software and code

Policy information about availability of computer code

Data collection	CryoEM data were collected with EPU 1.12.0.79 (Thermofisher Scientific). Mass spectrometry data was collected with Thermo Scientific Xcalibur 4.4.16.14 and ReFeyn Acquire 2.4.1
Data analysis	CryoEM data was analyzed with published softwares cryoEF (no version number available), RELION-3.1, CTFFIND 4-1, MotionCor2 (Relion 3.1 implementation), crYOLO 1.5.4, DeepEMhancer (0.13-cuda10), Coot 0.9-pre, ISOLDE 1.1.0, ChimeraX 1.1, Acedrg (222), REFMAC5, Phenix 1.18rc1-3769, Molprobity (no version number available), PyMOL 2.0, DataGraph (4.5.1). Mass spectrometry data was analyzed using MaxQuant (versions 1.5.3.30 and 1.6.10.0), pLink 2.3.9, RStudio 1.4.1717, ReFeyn Discover 2.4.2. Modeling of clusterin was performed using tRosetta (no version number available) and Modeller 9.25.

For manuscripts utilizing custom algorithms or software that are central to the research but not yet described in published literature, software must be made available to editors and reviewers. We strongly encourage code deposition in a community repository (e.g. GitHub). See the Nature Research guidelines for submitting code & software for further information.

Data

Policy information about availability of data

All manuscripts must include a data availability statement. This statement should provide the following information, where applicable:

- Accession codes, unique identifiers, or web links for publicly available datasets
- A list of figures that have associated raw data
- A description of any restrictions on data availability

Source data are provided with this paper. Data supporting the findings of this manuscript are available from the corresponding authors upon reasonable request. The MS raw data generated in this study have been deposited in the ProteomeXchange partner MassIVE database under the accession code MSV000087092. The output of the MS identification software is included as a Supplementary Data file. The processed MS data generated in this study underlying (Fig. 1c, Fig. 2b, Fig. 4e,

Supplementary Fig. 3b-d, Supplementary Fig. 4c-f, and Supplementary Fig. 6a-b) are included as a Source Data file. The cryo EM maps generated in this study have been deposited in the Electron Microscopy Data Bank under the accession codes EMD-12646, EMD-12647, EMD-12648, EMD-12649, EMD-12650, EMD-12651. The structural models generated in this study have been deposited in the Protein Data Bank under the accession codes 7NYC and 7NYD. Structural models used to initiate model building of sMAC were accessed from the Protein Data Bank under the accession codes 6H03, 6H04, 4A5W, 2WCY, and 6CXO. Structural models used in data analysis were accessed from the Protein Data Bank under the accession codes 1W33, 6SB3, 6SB5, 3NSJ, 4OEJ, and 5J68.

Field-specific reporting

Please select the one below that is the best fit for your research. If you are not sure, read the appropriate sections before making your selection.

☒ Life sciences ☐ Behavioural & social sciences ☐ Ecological, evolutionary & environmental sciences

For a reference copy of the document with all sections, see nature.com/documents/nr-reporting-summary-flat.pdf

Life sciences study design

All studies must disclose on these points even when the disclosure is negative.

Sample size	For structural studies, 1,637,429 particles were picked from 24,810 electron micrograph movies using an automated picking program that identifies particles based on a general neural network model as implemented in crYOLO or by using 2D class averages of sMAC particles in a template-based picking algorithm as implemented in RELION 3.1.
Data exclusions	For structural studies, electron micrograph movies with substantial drift and crystalline ice were excluded. sMAC picked particles were excluded based on 2D and 3D classification. Particles were removed from the dataset if their 2D class averages lacked obvious secondary structure elements. Particles were also removed from the dataset if they did not contribute to a reconstruction showing a clearly defined stoichiometry for C9 after 3D classification. Particles from over-represented views were removed to improve the anisotropy in the 3D reconstruction. Classes that were consistent with either 1, 2 or 3 copies of C9 were taken forward for further analysis.
Replication	XL-MS experiments were performed in triplicate, with 5% FDR and filter of 10 ppm used for the search. Only cross-links identified in all triplicates were included in further analysis. All attempts at replication were successful.
Randomization	Not relevant to this study, since samples were not allocated into experimental groups.
Blinding	Not relevant to this study, since there were no group allocations in this study.

Reporting for specific materials, systems and methods

We require information from authors about some types of materials, experimental systems and methods used in many studies. Here, indicate whether each material, system or method listed is relevant to your study. If you are not sure if a list item applies to your research, read the appropriate section before selecting a response.

Materials & experimental systems

n/a	Involved in the study
☒	☐ Antibodies
☒	☐ Eukaryotic cell lines
☒	☐ Palaeontology and archaeology
☒	☐ Animals and other organisms
☒	☐ Human research participants
☒	☐ Clinical data
☒	☐ Dual use research of concern

Methods

n/a	Involved in the study
☒	☐ ChIP-seq
☒	☐ Flow cytometry
☒	☐ MRI-based neuroimaging
